# Quantifying Annual Photon Absorption in 55 Bamboo Species: A Standardized Modeling Approach Using Peak-Season Leaf Optical Traits and Long-Term Radiation Data

**DOI:** 10.3390/plants15071105

**Published:** 2026-04-03

**Authors:** Changlai Liu, Mengxiao Wang, Fanfan He, Zhaoming Shi, Jianjun Zhang, Guohua Liu

**Affiliations:** 1 National Key Laboratory for the Development and Utilization of Forest Food Resources, Co-Innovation Centre for Sustainable Forestry in Southern China, Bamboo Research Institute, Nanjing Forestry University, Nanjing 210037, China; lcl2012@njfu.edu.cn (C.L.); 8231711755wmx@njfu.edu.cn (M.W.); 8231711769hff@njfu.edu.cn (F.H.); 3211700723@njfu.edu.cn (J.Z.); 2 Yibin Forestry and Bamboo Industry Research Institute, Yibin 644005, China; yblzyjyszm@163.com

**Keywords:** bamboo, light interception, leaf economic spectrum, photosynthetically active radiation, ecological adaptation

## Abstract

To accurately quantify the intrinsic absorption efficiency of bamboo leaves to the solar spectrum, we measured the reflectance and transmittance of leaves from 55 bamboo species cultivated at the same site, and developed a mathematical model to calculate the annual cumulative photon absorption of photosynthetically active radiation (PAR) per leaf. The results showed the following: (1) Bamboo leaf optical properties exhibited high instrumental and spatial measurement consistency, with transmittance not significantly fluctuating with changes in incident light intensity or quality. (2) Bamboo leaves exhibited significant spectral selective absorption characteristics, with stronger absorption of blue and red light and weaker absorption of green light; *Phyllostachys vivax* had the highest mean absorptance per unit area, while *Chimonobambusa tumidinoda* had the lowest. (3) The annual photon absorption per unit leaf area ranged from 1.83 × 10^5^ to 9.86 × 10^5^ μmol, with *Phyllostachys iridescens* being the lowest and *Chimonobambusa marmorea* the highest. The annual photon absorption per single leaf ranged from 1.84 × 10^6^ to 5.13 × 10^7^ μmol, with *Indocalamus decorus* achieving the highest total absorption due to its largest leaf area (114.9 cm^2^), while *Bambusa multiplex* var. *riviereorum* was the lowest. (4) All tested bamboo species showed consistent seasonal dynamics in photon absorption, with the highest in summer and lowest in winter. Although unit-area absorptance reflects the intrinsic light interception efficiency, leaf morphology has a substantial influence (explaining 99.56% of the variance) in determining total light acquisition per leaf.

## 1. Introduction

Light is a major environmental factor affecting plant growth and development, regulating plant photosynthesis, photomorphogenesis, and biomass allocation through variations in intensity, quality, and photoperiod. The light environment of natural habitats exhibits strong spatiotemporal heterogeneity [1]. In terms of time, the photosynthetic photon flux density (PPFD) undergoes complex dynamic fluctuations caused by seasonal changes, weather types and diurnal rhythms [2,3,4,5,6,7]. In terms of space, light quality varies according to geographical factors such as altitudinal gradients and atmospheric attenuation [8]. To determine bamboo light-use efficiency and to understand their light adaptation strategies, it is essential to physically resolve the characteristics of photosynthetically active radiation (PAR) across various temporal scales [9,10].

Bamboos are rapidly growing perennial clonal plants of the *Bambusoideae* subfamily that have become important global forest resources due to their remarkable environmental adaptability [11]. Various bamboo species have evolved unique photosynthetic apparatuses and morphological adaptation strategies to cope with diverse light environments [12,13]. Leaves are the organs that capture light energy directly [14,15]; their optical properties (reflectance, transmittance, and absorptance) determine the percentage of incident light actually intercepted and used for photochemical reactions, and thus they are the first step and an important intrinsic factor in determining the photosynthetic efficiency [16,17,18,19,20]. However, long-term quantification of total leaf light energy absorption in bamboo photosynthetic physiology has rarely been investigated.

We selected the experimental site, a representative bamboo-growing region in subtropical China, as the uniform background and constructed a high-precision regional solar spectrum and dynamic photon flux model based on continuous in situ observations over 2.5 years. Then, we measured the spectral reflectance and transmittance of 55 species of bamboo introduced to the site systematically, and calculated the total photon absorption of individual leaves in four seasons and the whole year under uniform light input conditions. The objective of this study is to eliminate the confounding effects of native environmental differences and to standardize the comparison of the light capture capacities of bamboo species at the energy input source. These results will provide a robust ecological basis for estimating the carbon sink function of bamboo forest ecosystems.

Based on the above background, we hypothesized that: (1) different bamboo species exhibit significant variation in annual photon absorption, and this variation is related to species’ ecological adaptation strategies; (2) among species with similar leaf areas, absorptance becomes the decisive factor for light capture variation, while leaf area is the primary driver of total light acquisition. To test these hypotheses, we measured leaf optical properties of 55 bamboo species and integrated them with a 2.5-year meteorological dataset to quantify annual photon absorption at the single-leaf level. Importantly, to establish a standardized baseline, leaf optical traits were measured exclusively during the peak growing season (June 2024). Our annual modeling assumes these intrinsic traits remain constant, thereby isolating interspecific differences from temporal plasticity.

## 2. Materials and Methods

### 2.1. Study Site Description

The experiments were performed in the Bamboo Germplasm Resource Garden of Nanjing Forestry University (118°49′11″ E, 32°04′48″ N), situated in a temperate northern subtropical humid climate zone, with a mean annual temperature of 15.4 °C, the hottest month (July) 28.1 °C, the coldest month (January) 2.7 °C, an annual temperature range of 25.3 °C, annual precipitation of 1090.4 mm (more than 40% occurs between June and August), a mean annual relative humidity of 76%, and an annual sunshine duration of 1980 h. The soil is typical yellow-brown earth with a pH of 5.5–6.5 and organic matter content of 1.5–2.8%, characterizing good water and fertilizer retention capabilities.

### 2.2. Measurement and Collection of Solar Spectral Data and Meteorological Records

Field measurements of solar spectral irradiance (230–850 nm wavelength range, 1 nm resolution) were conducted from September 2022 to June 2025 using a UV spectral irradiance meter (OHSP-350UVP, Hangzhou Hopoo Light&Color Technology Co., Ltd., Hangzhou, China). To eliminate subjective sampling bias and accurately capture seasonal radiation dynamics, representative measurement days were strictly anchored to key astronomical solar terms: the Vernal Equinox, Summer Solstice, Autumnal Equinox, and Winter Solstice (or the nearest equivalent dates if the exact days experienced non-target weather). On these selected dates, continuous full-day monitoring was performed from sunrise to sunset at one-hour intervals to acquire complete diurnal spectral curves across typical weather conditions (sunny, cloudy, and rainy), with any occasional missing data points gap-filled via linear interpolation of adjacent hourly records. Furthermore, to determine the seasonal frequencies of different weather types required for the annual aggregation model, long-term meteorological records spanning a continuous period from 2014 to 2024 were obtained from the National Meteorological Information Center of China (https://data.cma.cn/). To eliminate subjective bias, the definitions of “sunny,” “cloudy,” and “rainy” days were directly adopted from the Center’s official classifications, which are rigorously defined by standard national meteorological criteria including daily cloud cover and cumulative precipitation.

### 2.3. Plant Material and Sampling

Three healthy adult individuals of each species were chosen, and fully expanded mature leaves (the 2nd to 3rd leaves from the shoot tip) were sampled from the southeast-facing healthy branches, placed in an insulated box with ice packs, and brought back to the laboratory for optical property measurements within 2 h.

In June 2024, leaf samples were taken from 55 species of bamboo (18 from *Phyllostachys*, 8 from *Bambusa*, 6 from *Chimonobambusa*, 6 from *Pleioblastus*, 3 from *Pseudosasa*, 3 from *Indosasa*, 2 from *Indocalamus*, 2 from *Oligostachyum*, and 1 each from 7 other genera; Table S1) during the growing season when the species were growing vigorously in the Bamboo Germplasm Resource Garden of Nanjing Forestry University.

### 2.4. Measurement of Leaf Optical Properties

#### 2.4.1. Reflectance and Transmittance

Leaf reflectance and transmittance in the photosynthetically active radiation range were measured using a spectrophotometer (CR7, Shenzhen 3nh Technology Co., Ltd., Shenzhen, China) and a color haze meter (YH1100, Shenzhen 3nh Technology Co., Ltd., Shenzhen, China), both with a wavelength interval of 10 nm, and a D65 standard light source with an 8° observation angle; the instruments were calibrated with standard white and black boards before measurement. For transmittance measurements, fresh leaves were placed flat to cover the measurement aperture completely, and spectral data for reflectance and transmittance were collected from the center of each leaf. To determine how light intensity affects transmittance, we measured at distances of 5 cm, 10 cm, 20 cm, 30 cm, and 40 cm from a stationary light source. Additionally, to assess the impact of light quality on transmittance, we selected four distinct light sources: natural sunlight (S), a 6000 K white LED (W), a 4000 K mixed yellow-white LED (M), and a 2000 K yellow LED (Y). All measurements were taken within 4 h of sampling, with laboratory conditions held at 25 ± 1 °C and 50 ± 5% relative humidity.

#### 2.4.2. Leaf Area

Leaf area was determined using digital image analysis. High-resolution digital images of the leaves were acquired using a flatbed scanner (Epson Perfection V19, Epson, Nagano, Japan), and leaf area was subsequently calculated using ImageJ 1.52a (NIH, Bethesda, MD, USA) software.

### 2.5. Modeling Annual Photon Absorption

We developed a semi-empirical model to quantify the Annual Absorbed Photons (AAP) by coupling leaf optical traits with the stochastic weather-driven radiation framework.

#### 2.5.1. Intrinsic Leaf Absorptance

Spectral absorptance [α(*λ*)] was calculated based on the conservation of energy principle:

α(λ)=1−[ρ(λ)+τ(λ)]

where α(*λ*) and τ(*λ*) are the spectral reflectance and transmittance at wavelength λ, respectively.

#### 2.5.2. Diurnal Photon Flux Integration

The daily photon absorption (*AP_s*, *w*) for a single leaf under a specific season (*s*) and weather type (*w*) was calculated by integrating the diurnal course of spectral irradiance:

$$AP_{s,w}=LA\cdot \sum_{i\in \{B,G,R\}}\left[{\overset{\text{-}}{\alpha }}_{i}\cdot \int_{t_{sunrise}}^{t_{sunset}}PPFD_{s,w,i}(t)\;dt\right]$$

where *LA* is the leaf area (m^2^). *i* represents the wavebands: Blue (400–500 nm), Green (500–600 nm), and Red (600–700 nm). *αi* is the mean leaf absorptance for waveband i. ∫ *PPFDs*, *w*, *i*(*t*) dt represents the daily cumulative photon flux density (μmol m^−2^) derived from the area under the diurnal curve observed in Nanjing.

#### 2.5.3. Annual Aggregation Model

The total Annual Absorbed Photons (AAP, μmol leaf^−1^ year^−1^) were computed by weighting the daily absorption by the seasonal frequency of weather types:

$$AAP=\sum_{s=1}^{4}\sum_{w=1}^{3}(D_{s}\cdot P_{s,w}\cdot AP_{s,w})$$

where *Ds* is the total number of days in seasons. *Ps*, *w* is the empirical probability (frequency) of weather type w occurring in season s, derived from long-term meteorological records.

### 2.6. Statistical Analysis

Data were organized and preliminarily calculated using Microsoft Excel 2019 (Microsoft Corporation, Redmond, WA, USA). Data visualization and statistical analyses were performed using R software (version 4.5.2; R Core Team, Vienna, Austria). The normality of the data distribution was assessed using the Shapiro–Wilk test, and homogeneity of variances was verified using Levene’s test. One-way analysis of variance (ANOVA) was used to test for differences among groups, with significance set at *p* < 0.05. Tukey’s honestly significant difference (HSD) test was employed for post hoc multiple comparisons when ANOVA results were significant. The diurnal variation curve of light intensity was fitted using second-order polynomial regression based on full-day solar spectral data, and the area under the curve was calculated to determine the daily cumulative photon count per unit area. All data are presented as mean ± standard deviation (SD) unless otherwise specified. To quantify the relative contributions of leaf area and absorptance to the interspecific variation in total annual absorbed photons (AAP), we performed a variance decomposition analysis following logarithmic transformation of the variables. This mathematically isolates the percentage of variance explained by leaf morphology versus intrinsic optical traits.

## 3. Results

### 3.1. Seasonal Dynamics of Solar Spectra at the Experimental Site

**Figure 1:**
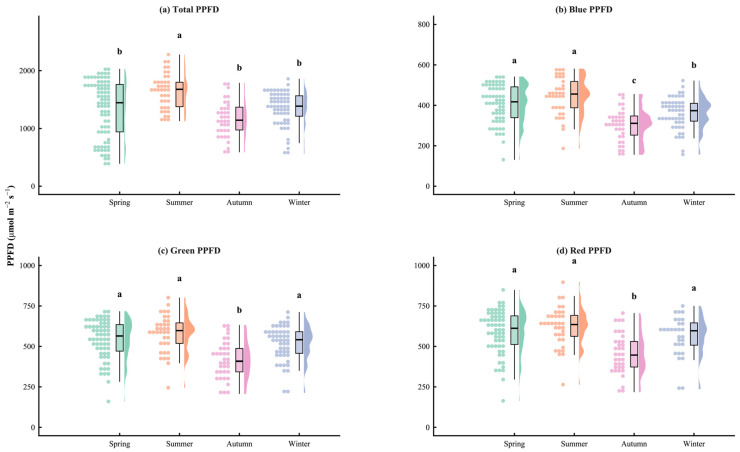
Comparison of PPFD in Four Seasons. Data are visualized using Raincloud plots, which combine raw data visualization (scatter dots, left), probability density estimation (half-violin plots, right), and descriptive statistics (boxplots, center). In the boxplots, the thick horizontal line represents the median, the box limits indicate the interquartile range (IQR), and the whiskers extend to 1.5 × IQR. The panels display (**a**) Total PPFD, (**b**) Blue PPFD, (**c**) Green PPFD, and (**d**) Red PPFD. Colors represent seasons: Spring (green), Summer (orange), Autumn (pink), and Winter (blue). Different lowercase letters above the plots indicate statistically significant differences between seasons at the *p* < 0.05 level (determined by one-way ANOVA followed by Tukey’s HSD test). Note: *Y*-axis scales vary among panels to appropriately display the intensity range of each spectral band.

Based on solar spectral data gathered at the experimental site between September 2022 and June 2025, seasonal variations in total PPFD and its blue, green, and red components were examined. The results clearly displayed a seasonal pattern (Figure 1) with the following intensity order—Summer > Spring > Winter > Autumn—which was primarily driven by the higher frequency of rainy days in autumn and sunny days in winter (Table 1). In summer, the total PPFD was between 1125.9 and 2277.0 μmol·m^−2^·s^−1^, and in winter, it was between 556.2 and 1857.9 μmol·m^−2^·s^−1^. According to the frequency of weather conditions, summer had the most cloudy (28.99 percent) and rainy (58.33 percent) days, while winter had the most sunny days (34.69 percent) (Table 1). The amount of daylight varied from a minimum of 10 h and 29 min in the winter to a maximum of 13 h and 51 min in the summer.

**Figure 2:**
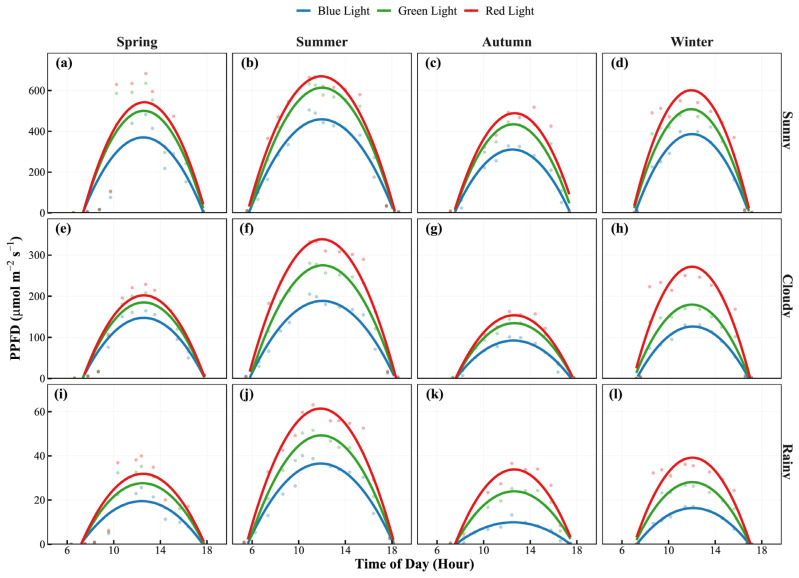
Diurnal dynamics of photosynthetic photon flux density (PPFD) for different light qualities across four seasons and three weather conditions. The scatter points represent the observed values, and the solid lines represent the fitted curves using second-order polynomial regression. Colors indicate different wavebands: blue lines for blue light, green lines for green light, and red lines for red light. The panels are organized by weather conditions (Rows: Sunny, Cloudy, Rainy) and seasons (Columns: Spring, Summer, Autumn, Winter). Subplots (**a**–**l**) correspond to specific season–weather combinations. Note that the scales of the *Y*-axis vary across rows to accommodate the significant differences in light intensity under different weather conditions.

Based on continuous monitoring, we modeled the diurnal PPFD dynamics under typical weather conditions (Figure 2). The cumulative daily photon flux per unit area peaked on sunny summer days (Blue: 1.34, Green: 1.81, Red: 2.02 × 10^7^ μmol·m^−2^) and reached a minimum on rainy autumn days (Table S2). Under identical seasonal and weather conditions, photon flux followed the order: Red > Green > Blue. Weather conditions significantly impacted light quantity; for instance, transitioning from sunny to cloudy weather in autumn reduced photon flux by approximately 60–70%.

### 3.2. Measurement Consistency of Leaf Optical Properties

**Figure 3:**
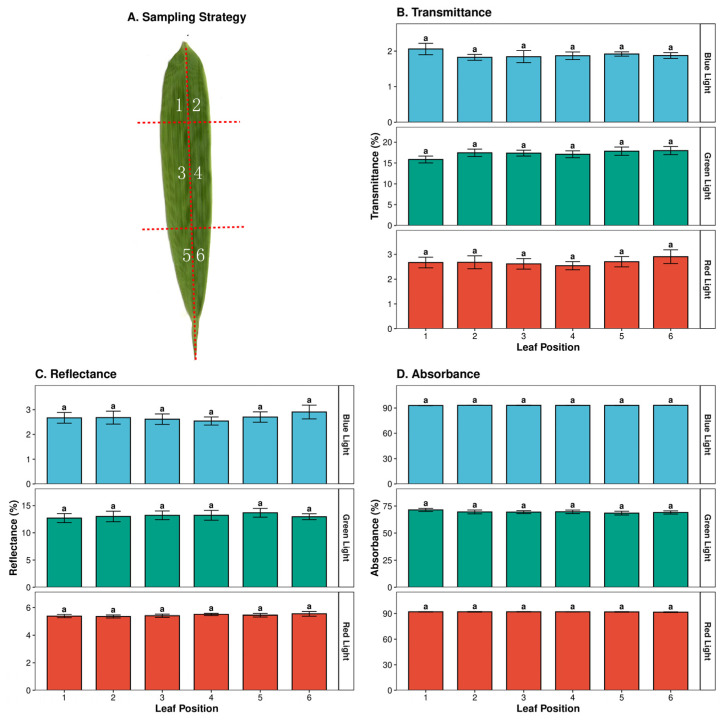
Leaf spectral reflectance, transmittance, and absorbance characteristics across different sampling positions. (**A**) Schematic representation of the sampling strategy, dividing the leaf blade into six specific regions (Positions 1–6) for optical measurements. (**B**–**D**) Statistical analysis of leaf transmittance (%) (Panel (**B**)), reflectance (%) (Panel (**C**)), and absorbance (%) (Panel (**D**)) in the Blue, Green, and Red wavebands across the six leaf positions. Data are presented as mean ± standard deviation (SD). Different lowercase letters above the bars indicate significant differences (*p* < 0.05) based on one-way ANOVA followed by Tukey’s HSD post hoc test. The presence of the same letter (‘a’) indicates no statistically significant difference among the different leaf positions.

To assess the homogeneity of leaf optical properties, *Chimonobambusa quadrangularis* leaves were divided into six regions and measured. No significant differences (*p* > 0.05) were found in transmittance, reflectance, or absorptance across the different leaf positions (Figure 3).

**Figure 4:**
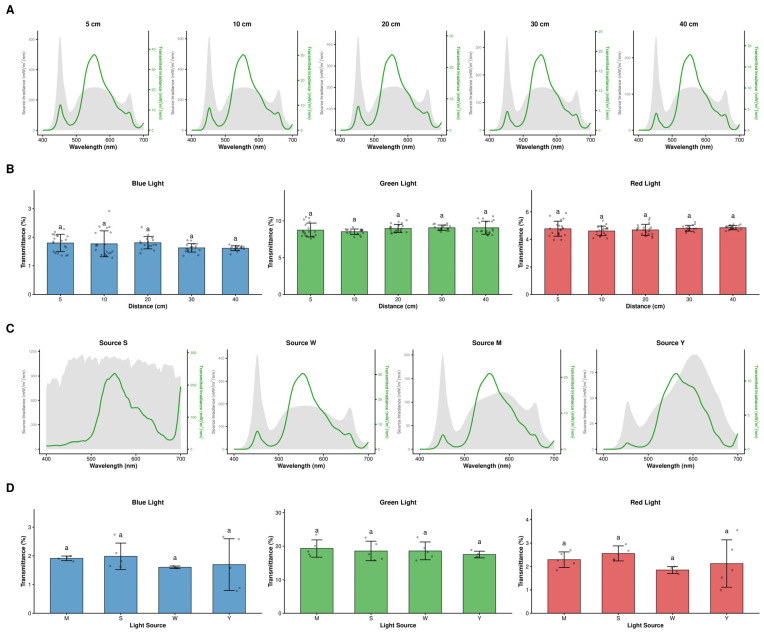
Effects of light intensity (distance) and light quality (source) on leaf spectral transmittance characteristics. (**A**,**C**) Spectral distribution profiles of source irradiance (gray shaded area, left axis) and transmitted irradiance through leaves (green line, right axis), measured at 5, 10, 20, 30, and 40 cm from the light source (Panel (**A**)) and under four different light sources (Source S, Source W, Source M, and Source Y, Panel (**C**)). (**B**,**D**) Statistical analysis of leaf transmittance (%) in the Blue, Green, and Red wavebands; Panel (**B**) compares transmittance at different distances; Panel (**D**) compares transmittance under different light sources. Data are presented as mean ± standard deviation (SD) with individual data points overlaid. Different lowercase letters above the bars indicate significant differences (*p* < 0.05) based on one-way ANOVA followed by Tukey’s HSD post hoc test. The presence of the same letter (‘a’) indicates no statistically significant difference among treatments.

In addition, we tested the stability of leaf transmittance with changes in light intensity (5 to 40 cm distance) and light quality (four different light sources). Although source irradiance decreased dramatically with distance, the spectral shape of leaf transmittance remained stable (Figure 4A). Statistical analysis showed that leaf transmittance for blue (1.57% to 1.80%), green (8.56% to 8.93%), and red (4.56% to 4.78%) wavebands did not vary significantly with light intensity (Figure 4B) or light source quality (*p* > 0.05) (Figure 4D), which indicates that bamboo leaf optical properties exhibited high spatial and instrumental measurement consistency.

### 3.3. Interspecific Differences in Leaf Optical Properties

**Figure 5:**
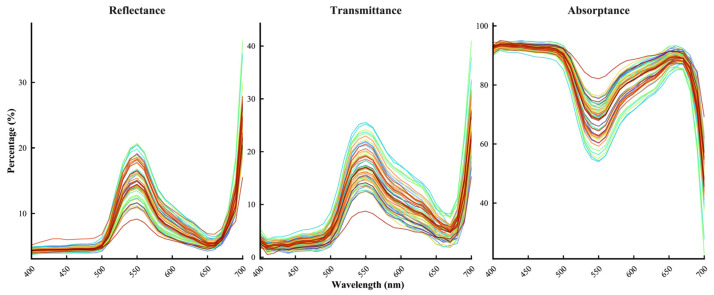
Spectral profiles of leaf optical properties for 55 bamboo species across the visible light spectrum (400–700 nm). The panels display leaf reflectance (**left**), transmittance (**middle**), and absorptance (**right**). Each colored line represents the mean spectral curve of a single bamboo species (*n* = 10). Different colors are used to distinguish among the 55 species.

The 55 bamboo species showed similar spectral trends in the 400–700 nm range (Figure 5): Reflectance: Low in the blue region (~5%), peaked in the green region (~550 nm), and showed a valley in the red region (670–680 nm); *Phyllostachys vivax* had the lowest mean reflectance, while *Chimonobambusa tumidinoda* had the highest. Transmittance: Followed a pattern similar to reflectance, with a peak at 550 nm; *P. vivax* had the lowest mean transmittance, while *C. tumidinoda* had the highest. Absorptance: Bamboo leaves had high absorptance in the blue (~92%) and red regions, with a clear “green valley” at 550 nm; in contrast to reflectance and transmittance, *C. tumidinoda* showed the lowest mean absorptance, while *P. vivax* displayed the highest. Furthermore, in the red light band, *Oligostachyum sulcatum* had the lowest absorptance, while *P. vivax* maintained the highest.

### 3.4. Annual Photon Capture by Bamboo Leaves

By integrating leaf optical properties with the local light environment model, we calculated the annual absorbed photons (AAP) for single leaves of the 55 species (Table S3). All species showed distinct seasonal dynamics, with photon absorption being highest in summer (28.0–29.4% of total) and lowest in winter (21.9–23.2%) (*p* < 0.05).

Significant interspecific variation was observed in annual photon absorption:

Per unit leaf area (AParea): Ranged from 1.83 × 10^5^ to 9.86 × 10^5^ μmol·cm^−2^. *Phyllostachys iridescens* was the lowest, while *Chimonobambusa marmorea* (Mitford) Makino f. *variegata* (Makino) Ohwi was the highest.

Per single leaf (AAP): Ranged from 1.84 × 10^6^ to 5.13 × 10^7^ μmol. *Indocalamus decorus* achieved the highest total absorption (approx. 17.5 times that of the lowest, *Bambusa multiplex* var. *riviereorum*).

**Figure 6:**
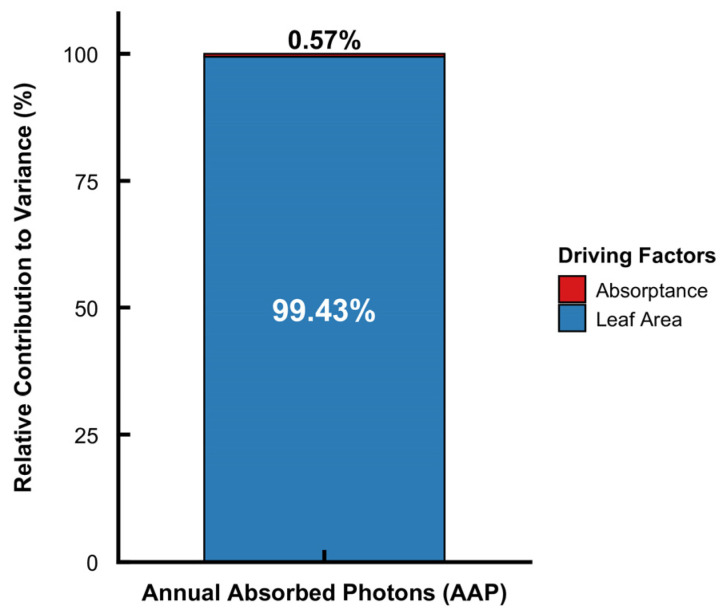
Variance decomposition of annual absorbed photons (AAP) based on the Lindeman–Merenda–Gold (LMG) method. The stacked bar chart illustrates the relative contributions of leaf area (blue) and unit-area absorptance (red) to the interspecific variance in total photon acquisition across the 55 bamboo species. Prior to the multiple linear regression, the variables were logarithmically transformed to convert their multiplicative relationship into an additive model framework.

To quantitatively partition the relative contributions of morphological and optical traits across all 55 species, a variance decomposition analysis was performed based on multiple linear regression. The results revealed that leaf area explained 99.56% of the interspecific variance in total AAP, while unit-area absorptance accounted for the remaining 0.44% (Figure 6). This statistically confirms that leaf morphology acts as the overwhelmingly primary driver of total light acquisition at the single-leaf level. Nevertheless, among species with similar leaf areas, intrinsic absorptance becomes the decisive factor modulating total photon capture. For instance, although *Pseudosasa amabilis* (48.5 cm^2^) and *Pseudosasa japonica* (51.2 cm^2^) have similar leaf sizes, the higher absorptance of *P. amabilis* resulted in significantly higher annual photon capture (1.90 × 10^7^ μmol) compared to *P. japonica* (1.78 × 10^7^ μmol).

## 4. Discussion

### 4.1. Ecological and Physiological Mechanisms of Photon Absorption in Bamboo

In this study, we analyzed light environment variation based on continuous monitoring at the experimental site of light environment data, and the results indicated that seasonal weather dynamics and diurnal cycles drive large-scale variations in sunshine duration and annual cumulative solar radiation. Despite the fact that the 55 bamboo species used in this study have been introduced and grown in the same habitat in Nanjing for almost 40 years, the AAP per single leaf still showed large interspecific variation (1.84 × 10^6^ to 5.13 × 10^7^ μmol), which indicated that bamboos as gramineous plants have not only conserved leaf optical properties during long evolutionary history [21], but also developed highly diverse environmental adaptation strategies [22].

The visible light band absorption of all tested bamboo species exhibited a strong and consistent “two peaks and one valley” absorption pattern, with extremely high absorptance (~92%) in the blue (400–500 nm) and red (600–700 nm) bands, and a distinct absorption valley in the green band (500–600 nm), with a “green peak” in both transmittance and reflectance (Figure 5) [23]. This spectral selectivity is determined by the physical properties of photosynthetic pigments, which have strong characteristic absorption bands in the red and blue regions [24]. The photons captured by the antenna pigments directly drive not only photochemical reactions but also photomorphogenesis [25]. Light significantly influences leaf area development. For example, blue light suppresses leaf expansion: increasing the blue light ratio reduces plant height and alters the morphological development of cucumber and tomato [26]. Similarly, in lettuce, cucumber, and tomato, increasing the blue light fraction from 10% to 30% consistently decreased leaf area and dry mass, suggesting that blue light acts as a potent signal for compact growth [27]. Conversely, higher proportions of red light generally promote leaf expansion. In *Salvia plebeia*, leaf area was greatest under pure red light (R10:B0) and the R7:B3 treatment [28]. Rocket salad exhibited larger leaf areas under R:B ratios ≥ 2 compared to lower ratios. Notably, optimal R:B ratios for maximizing leaf area differ substantially among species. For example, cucumber grown under 100% blue light exhibited the largest leaf area, contrasting with the typical pattern. An intermediate R:B ratio (typically 3:1 to 5:1) often balances expansion with normal morphology [26], but empirical determination for each species remains essential. Specifically, the seasonal shifts in the red/blue (R/B) light ratio and the elevated absolute blue light intensity during summer (Figure 1 and Figure 2) act as crucial environmental signals regulating cellular elongation. This dynamic spectral quality likely restricts excessive leaf expansion under peak irradiance, synergistically shaping the diverse, species-specific leaf areas that ultimately determine total single-leaf photon acquisition. However, the relatively low single-pass absorptance in the green band does not mean this light is “ineffective”; rather, because less green light is absorbed by the leaf surface, it will be reflected and refracted multiple times, which is referred to as the detour effect [29], resulting in a greatly increased optical path length [30,31]. In addition, for dense bamboo forests with generally high LAI, the higher transmittance at 550 nm enables more green light to be transmitted through the top canopy, which is referred to as the sieve effect [29], thereby supplementing the photosynthetically active radiation for leaves in the middle and lower layers, avoiding premature light saturation or photoinhibition in upper leaves [32], and maintaining the overall LUE of the canopy.

The interspecific variation in AAP reflects the ecological niche differentiation of bamboo species along the Leaf Economics Spectrum (LES) [33,34,35]; high-AAP species, such as *Chimonobambusa marmorea*, mainly originated from understory habitats in low-light conditions, which often are hypothesized to correlate with specialized leaf anatomical structures, such as the presence of fusoid cells (unique to the Bambusoideae subfamily, which are mainly distributed on either side of the vascular bundle, filled with gas, and enhance the lateral scattering and diffuse reflection of incident light within the mesophyll tissue to increase the optical path length and photon interception rate in weak light environments), and a high SLA, which tends to maximize their light interception capacity [36,37,38,39].

On the other hand, the low-AAP species, which are mainly represented by large running bamboos such as *Phyllostachys iridescens*, evolved in full-light habitats above the canopy [40]. The relatively lower absorptance is, in fact, an active morphological–physiological synergistic photoprotection mechanism [41]; the leaf epidermis of these bamboos often develops a thick cuticle or a dense wax crystal structure to actively resist excessive light energy input by increasing specular reflection [42,43]. Simultaneously, they might potentially elevate the concentration of photoprotective pigments, such as carotenoids, in their internal mesophyll tissues to enhance NPQ [44], thereby avoiding the irreversible oxidative damage to the core proteins of PSII by safely dissipating excess light energy as heat. This “high reflection–low absorption” strategy theoretically trades off some potential light energy capture but significantly increases the survival resistance and conservative adaptability of the plants under combined high-light and high-temperature stress [45,46].

### 4.2. Methodological Limitations and Future Perspectives

While this study provides a standardized framework for comparing the intrinsic light-capture capacities of 55 bamboo species, certain methodological constraints should be acknowledged. First, to establish a unified baseline and eliminate confounding environmental variables, leaf optical traits were measured at a single time point during the vigorous growing season (June 2024). Although mature evergreen bamboo leaves exhibit relative temporal stability in their optical properties once fully expanded, this static approach does not account for potential seasonal plasticity or ontogenetic shifts. Thus, our model calculates the intrinsic potential for annual photon absorption under standardized conditions rather than real-time dynamic acquisition. Second, leaf transmittance was measured using a color haze meter rather than an integrating sphere. While highly efficient for high-throughput screening, it may inherently underestimate a fraction of diffuse transmitted light. However, because all species were measured under identical optical geometry, this systematic effect applies uniformly, keeping interspecific relative rankings highly robust.

The model estimates reported in this study are largely based on the single-leaf level under full-light conditions and have not completely accounted for the interactions of three-dimensional canopy structure, dynamic leaf inclination angles, and mutual leaf shading on the actual population light interception [47]. Future studies should incorporate more sophisticated canopy structure models and three-dimensional radiative transfer models to make a quantitative leap in the prediction of light absorption from the single-leaf to the canopy scale. Furthermore, while this study showed that interspecific variations in leaf absorptance are substantial, the micro-mechanisms underlying these variations, such as 3D mesophyll topology, and photosynthetic biochemical capacity, need to be systematically quantified. Specifically, utilizing the obtained high-resolution spectral data for the non-destructive inversion of pigment stoichiometry (such as chlorophyll and carotenoids) represents a highly promising and logical next step for future research. Establishing the theoretical photon absorption model proposed in this study with real physiological parameters, including biomass accumulation, photosynthetic carbon assimilation efficiency, and NPQ, will open new avenues for fundamental research into the mechanisms of light adaptation, and it will also be a good scientific basis for the accurate screening of high-light-efficiency bamboo germplasm and the targeted cultivation of carbon sink functions in bamboo ecosystems.

## 5. Conclusions

We established a mathematical model combining continuous meteorological monitoring and leaf optical traits to calculate annual absorbed photons (AAP) of 55 bamboo species in a uniform environment. Our main findings are as follows: (1) Bamboo leaf optical properties remained stable across different light intensities and qualities, exhibiting a consistent “two peaks and one valley” absorption pattern with high absorptance in blue (~92%) and red spectral bands. (2) Although unit-area absorptance represents the inherent light-interception efficiency of leaf tissues, leaf morphology, especially leaf area, has a major influence (explaining 99.56% of the variance) on the total light energy acquisition of a single leaf. (3) Significant interspecific variation in AAP (1.84 × 10^6^ to 5.13 × 10^7^ μmol) indicates that bamboos have evolved different ecological adaptation strategies along the Leaf Economics Spectrum. High-AAP species (e.g., *Chimonobambusa marmorea*) tend to maximize light interception capacity through specialized leaf anatomical structures such as fusoid cells, while low-AAP species (e.g., *Phyllostachys iridescens*) are hypothesized to employ photoprotection mechanisms including increased reflectance and non-photochemical quenching. These findings provide a theoretical basis and practical guidance for the optimization of bamboo forest structures, screening of shade-tolerant species, and prediction of the carbon sink potential of bamboo ecosystems.

## Figures and Tables

**Table 1:** The proportions of three types of weather in different seasons.

Weather	Spring	Summer	Autumn	Winter
Sunny	20.29%	12.68%	34.43%	34.69%
Cloudy	26.45%	28.99%	23.81%	22.51%
Rainy	53.26%	58.33%	41.76%	42.80%

## Data Availability

The data presented in this study are available on request from the corresponding author.

## Supplementary Materials

The following supporting information can be downloaded at: https://www.mdpi.com/article/10.3390/plants15071105/s1. Table S1: Detailed information on the bamboo plant materials; Table S2: Seasonal variations in cumulative photon flux density under different weather conditions; Table S3: Absorbed photon numbers of single leaves of different bamboo species.

## Abbreviations

The following abbreviations are used in this manuscript:

**Abbreviation/Symbol**	**Definition/Description**	**Unit**
Abbreviations		
PAR	Photosynthetically active radiation (400–700 nm)	–
PPFD	Photosynthetic photon flux density	μmol m^−2^ s^−1^
VIS	Visible spectrum radiation	–
UV	Ultraviolet radiation	–
LA	Leaf area	cm^2^
SLA	Specific leaf area	cm^2^ g^−1^
LES	Leaf economics spectrum	–
NPQ	Non-photochemical quenching	–
AAP	Annual absorbed photons (per single leaf)	μmol leaf^−1^ year^−1^
Symbols		
*λ*	Wavelength of light	nm
*α*	Leaf absorptance (fraction of light absorbed)	% or dimensionless
*ρ* (or *r*)	Leaf reflectance	% or dimensionless
*τ* (or *t*)	Leaf transmittance	% or dimensionless
*AP*	Daily absorbed photons	μmol leaf^−1^ day^−1^
*A*	Cumulative daily solar photons per unit area (Area under PPFD curve)	μmol m^−2^ day^−1^
*D*	Number of days in a specific season	days
*P*	Frequency/Probability of a specific weather type	%
Subscripts		
*b*, *g*, *r*	Wavebands: Blue (400–500 nm), Green (500–600 nm), Red (600–700 nm)	–
*s*	Season (Spring, Summer, Autumn, Winter)	–
*w*	Weather type (Sunny, Cloudy, Rainy)	–
area	Per unit leaf area	–
