# Supplementary material for: Quantifying Annual Photon Absorption in 55 Bamboo Species: A Standardized Modeling Approach Using Peak-Season Leaf Optical Traits and Long-Term Radiation Data
Source: Plants (Basel). 2026 Apr 3;15(7):1105. doi: 10.3390/plants15071105 (PMC13074584; doi:10.3390/plants15071105)
Supplement: Supplementary file 1 [file plants-15-01105-s001.zip › plants-4218876-supplementary.pdf]

Table S1. Detailed information on the bamboo plant materials.

No.	Scientific Name	Species Code	Genus
1	*Bashania fargesii*	Bfa	*Bashania*
2	*Drepanostachyum microphyllum*	Dmi	*Drepanostachyum*
3	*Indosasa shibataeoides*	Ish	*Indosasa*
4	*Pleioblastus maculatus*	Pma	*Pleioblastus*
5	*Phyllostachys bambusoides*	Pbl	*Phyllostachys*
6	*Pseudosasa amabilis* var. *tenuis*	Pst	*Pseudosasa*
7	*Pseudosasa amabilis*	Pam	*Pseudosasa*
8	*Chimonobambusa neopurpurea*	Cne	*Chimonobambusa*
9	*Pleioblastus gramineus*	Pgr	*Pleioblastus*
10	*Phyllostachys glauca*	Pgl	*Phyllostachys*
11	*Brachystachyum densiflorum*	Bde	*Brachystachyum*
12	*Shibataea chinensis*	Sch	*Shibataea*
13	*Chimonobambusa quadrangularis*	Cqa	*Chimonobambusa*
14	*Pleioblastus fortunei 'Feilüzhu'*	Pff	*Pleioblastus*
15	*Bambusa multiplex*	Brf	*Bambusa*
16	*Indosasa gigantea*	Igi	*Indosasa*
17	*Phyllostachys prominens*	Ppr	*Phyllostachys*
18	*Bambusa multiplex* var. *riviereorum*	Bmu	*Bambusa*
19	*Phyllostachys heterocycla*	Phe	*Phyllostachys*
20	*Phyllostachys bambusoides*	Pba	*Phyllostachys*
21	*Chimonobambusa marmorea*	Cbm	*Chimonobambusa*
22	*Phyllostachys iridescens*	Pir	*Phyllostachys*
23	*Chimonobambusa marmorea* (Mitford) Makino f. *variegata* (Makino) Ohwi	Cma	*Chimonobambusa*
24	*Bambusa multiplex* f. *'Fujian'*	Bmf	*Bambusa*
25	*Phyllostachys arcana 'Luteosulcata'*	Pal	*Phyllostachys*
26	*Phyllostachys aureosulcata aureocaulis*	Paa	*Phyllostachys*
27	*Phyllostachys vivax*	Pva	*Phyllostachys*
28	*Phyllostachys aurea* f. *holochrysa*	Pah	*Phyllostachys*
29	*Neosinocalamus affinis*	Nav	*Neosinocalamus*
30	*Phyllostachys spectabilis*	Psp	*Phyllostachys*
31	*Phyllostachys aureosulcata*	Pap	*Phyllostachys*
32	*Phyllostachys nigra* var. *henonis*	Pnh	*Phyllostachys*
33	*Phyllostachys edulis*	Ped	*Phyllostachys*
34	*Indocalamus decorus*	Ide	*Indocalamus*
35	*Phyllostachys mannii*	Pmg	*Phyllostachys*
36	*Bambusa multiplex* f. *'Nanlin'*	Bmn	*Bambusa*
37	*Pleioblastus argenteostriatus*	Par	*Pleioblastus*
38	*Chimonobambusa tumidinoda*	Ctu	*Chimonobambusa*
39	*Indocalamus tessellatus*	Ite	*Indocalamus*
40	*Pleioblastus sanmingensis*	Psa	*Pleioblastus*
41	*Oligostachyum sulcatum*	Osu	*Oligostachyum*
42	*Pseudosasa japonica*	Pja	*Pseudosasa*
43	*Oligostachyum lubricum*	Olu	*Oligostachyum*
44	*Sinobambusa tootsik*	Sto	*Sinobambusa*
45	*Phyllostachys vivax* McClure	Pvm	*Phyllostachys*
46	*Bambusa ventricosa*	Be	*Bambusa*
47	*Bambusa multiplex*	Bra	*Bambusa*
48	*Bambusa multiplex*	Bmr	*Bambusa*
49	*Semiarundinaria fastuosa*	Sfa	*Semiarundinaria*
50	*Bambusa rigida*	Bri	*Bambusa*
51	*Chimonobambusa sichuanensis*	Csi	*Chimonobambusa*
52	*Phyllostachys praecox*	Ppx	*Phyllostachys*
53	*Pleioblastus simonii*	Psi	*Pleioblastus*
54	*Indosasa sinica*	Isi	*Indosasa*
55	*Phyllostachys nigra*	Pnm	*Phyllostachys*

Table S2. Seasonal variations in cumulative photon flux density under different weather conditions.

Season	Sunny (S)			Cloudy (C)			Rainy (R)		
	Blue	Green	Red	Blue	Green	Red	Blue	Green	Red
Spring	0.88	1.21	1.32	0.36	0.45	0.49	0.05	0.07	0.08
Summer	1.34	1.81	2.02	0.55	0.81	1.02	0.11	0.15	0.19
Autumn	0.73	1.06	1.22	0.21	0.32	0.36	0.02	0.06	0.08
Winter	0.89	1.2	1.44	0.29	0.43	0.65	0.04	0.07	0.09

Table S3. Absorbed photon numbers of single leaves of different bamboo species.

No.	Species code	Leaf Area(cm2)	APspr	APsum	APaut	APwin	AParea	AAP
1	Bfa	39.29	3.31	4.66	3.78	4.60	0.42	16.40
2	Dmi	12.95	1.02	1.44	1.17	1.42	0.39	5.06
3	Ish	28.23	2.23	3.14	2.54	3.10	0.39	11.00
4	Pma	22.66	1.88	2.65	2.14	2.61	0.41	9.29
5	Pbl	17.89	1.48	2.08	1.69	2.06	0.41	7.31
6	Pst	42.99	3.59	5.06	4.10	4.99	0.41	17.70
7	Pam	48.47	3.84	5.40	4.37	5.33	0.39	18.90
8	Cne	35.40	3.07	4.32	3.51	4.27	0.43	15.20
9	Pgr	8.35	0.73	1.03	0.84	1.02	0.43	3.62
10	Pgl	15.13	1.30	1.84	1.49	1.81	0.43	6.44
11	Bde	34.84	2.92	4.12	3.34	4.07	0.42	14.40
12	Sch	15.73	1.36	1.92	1.55	1.89	0.43	6.72
13	Cqa	23.24	1.96	2.77	2.24	2.73	0.42	9.70
14	Pff	23.89	2.18	3.07	2.49	3.03	0.45	10.80
15	Brf	5.21	0.47	0.66	0.53	0.65	0.44	2.31
16	Igi	26.86	2.34	3.30	2.67	3.25	0.43	11.60
17	Ppr	23.49	2.03	2.86	2.32	2.82	0.43	10.00
18	Bmu	4.19	0.37	0.52	0.42	0.52	0.44	1.83
19	Phe	7.06	0.60	0.85	0.69	0.84	0.42	2.96
20	Pba	19.61	1.73	2.44	1.98	2.41	0.44	8.55
21	Cbm	8.20	0.68	0.96	0.78	0.95	0.41	3.36
22	Pir	17.77	0.66	0.92	0.75	0.91	0.18	3.24
23	Cma	8.05	1.60	2.25	1.83	2.22	0.98	7.90
24	Bmf	17.49	1.57	2.21	1.79	2.19	0.44	7.76
25	Pal	14.60	1.27	1.80	1.46	1.77	0.43	6.30
26	Paa	8.05	0.71	0.99	0.81	0.98	0.43	3.48
27	Pva	14.61	1.23	1.73	1.40	1.71	0.42	6.08
28	Pah	8.81	1.39	1.97	1.59	1.94	0.78	6.90
29	Nav	15.41	0.94	1.33	1.07	1.31	0.30	4.65
30	Psp	10.86	1.15	1.62	1.31	1.60	0.52	5.68
31	Pap	13.34	0.75	1.05	0.85	1.04	0.28	3.69
32	Pnh	9.57	0.78	1.10	0.89	1.09	0.40	3.86
33	Ped	7.67	0.64	0.90	0.73	0.88	0.41	3.14
34	Ide	114.93	10.30	14.50	11.80	14.30	0.44	51.00
35	Pmg	9.87	0.86	1.21	0.98	1.19	0.43	4.24
36	Bmn	13.74	1.24	1.74	1.41	1.72	0.45	6.12
37	Par	13.80	1.22	1.72	1.40	1.70	0.44	6.04
38	Ctu	7.57	0.59	0.83	0.67	0.82	0.39	2.91
39	Ite	38.51	3.39	4.77	3.87	4.71	0.44	16.70
40	Psa	26.30	2.18	3.07	2.49	3.04	0.41	10.80
41	Osu	13.27	1.05	1.47	1.19	1.45	0.39	5.16
42	Pja	51.22	4.52	6.37	5.16	6.29	0.44	22.30
43	Olu	16.28	1.45	2.04	1.66	2.02	0.44	7.16
44	Sto	24.59	2.20	3.11	2.52	3.07	0.44	10.90
45	Pvm	19.82	1.83	2.58	2.10	2.55	0.46	9.07

No.	Species code	Leaf Area(cm2)	APspr	APsum	APaut	APwin	AParea	AAP
46	Be	28.46	2.57	3.63	2.94	3.58	0.45	12.70
47	Bra	15.76	1.34	1.89	1.53	1.87	0.42	6.64
48	Bmr	12.60	1.13	1.60	1.30	1.58	0.45	5.61
49	Sfa	22.98	1.98	2.78	2.25	2.74	0.43	9.75
50	Bri	35.76	3.17	4.46	3.62	4.41	0.44	15.70
51	Csi	30.10	2.69	3.79	3.07	3.74	0.44	13.30
52	Ppx	20.43	1.78	2.51	2.04	2.48	0.43	8.82
53	Psi	24.12	2.00	2.81	2.28	2.78	0.41	9.87
54	Isi	34.89	2.98	4.19	3.40	4.14	0.42	14.70
55	Pnm	11.58	0.97	1.36	1.10	1.34	0.41	4.77
